# Mineralisation of atmospheric aerosol particles and further analysis of trace elements by inductively coupled plasma-optical emission spectrometry

**DOI:** 10.1016/j.mex.2017.05.002

**Published:** 2017-06-13

**Authors:** Aurélie Dufour, Christophe Migon

**Affiliations:** a Sorbonne Universités, UPMC Université Paris 06, UMR 7093, LOV, Observatoire Océanologique, F06230, Villefranche sur mer, France; b CNRS, UMR 7093, LOV, Observatoire Océanologique, F06230, Villefranche sur mer, France

**Keywords:** Determination of trace elements in the atmospheric aerosol, Mineralisation protocol, Atmospheric aerosol, Trace metals, Inductively coupled plasma-optical emission spectrometry

## Abstract

Several protocols using different treatments (various mixtures of acids at different temperatures for mineralisation) or using several analysis instrumentations were tested with the aim to define the method allowing the analysis of some groups of elements. This study proposes a protocol of sample treatment and analysis permitting in a single batch the determination of 16 elements (Al, As, Ba, Cd, Co, Cu, Fe, K, Mg, Mn, Na, Ni, Pb, Ti, V and Zn) with different chemical features such as volatile or refractory trace elements. This method is specifically adapted to chemical matrices found in unpolluted to moderately polluted atmospheric aerosol samples. Aerosol samples were digested using a mixture aqua regia/hydrofluoric acid at 130 °C during 2 h, and were then analysed with specifically tuned inductively coupled plasma-optical emission spectrometry.

• Reduction of costs: use of hot block, use of inductively coupled plasma-optical emission spectrometry (ICP-OES), easiness, reliability and adaptability to routine analysis
• Digestion of up to 54 samples at the same time in 2 h and low amount of material required, only 10 mg is necessary.
• Better accordance with Occupational Health and Safety requirements (reduced use of acids, in particular HF, no use of high-pressure Teflon bombs).

## Graphical abstract

**Figure:**
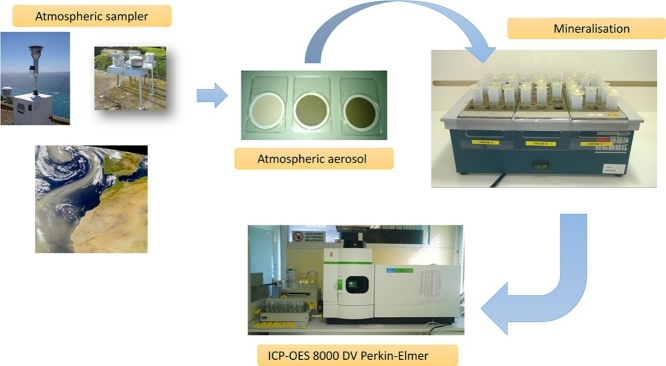

## Method details

The analysis of atmospheric aerosol samples collected on filters may sometimes be a difficult challenge, owing to the combined effects of chemically resistant matrices potentially present in the atmospheric aerosol (alumino-silicates, oxides, sea-spray, organic compounds [1]), low quantities of collected material (of the order of few milligrammes, typically) and trace/ultra-trace elemental concentrations encountered (of the order of few μg L^−1^, typically). Attempts to totally digest these matrices with a single protocol may lead to signal losses, therefore yielding low recoveries and hence underestimated measurements. Non-destructive analytical techniques such as X-ray fluorescence, proton-induced X-ray emission (PIXE) are currently being developed which skip the mineralisation phase, but their detection limits are still too high given the elemental concentrations found in the atmospheric aerosol: below 100 ng m^−3^, the degree of overestimation by Energy Dispersive X-ray Fluorescence (EDXRF) increases when concentrations decrease [2]. In addition, the non-uniformity of elemental distribution on filters may lead to erroneous results [3]. Therefore, mineralisation remains a crucial, and unavoidable phase in the preparation of natural particulate samples for elemental analysis.

The present work proposes an alternative, tentatively all-purpose mineralisation protocol (the splitting of samples into sub-samples by cutting filters is banned because of the non-uniform distribution of matter [4]), followed by an analytical method specifically adapted to trace elements in atmospheric particulate samples.

## Materials and methods

### Reagents, standards and materials

All reagents (HCl 30%, HNO_3_ 65%, HF 40%) were provided by Merck (Darmstadt, Germany), quality Suprapur^®^. Water was de-ionised by a Direct 8 Milli-Q^®^ Gradient System (Millipore), resistivity 18 ΜW cm. Calibrations were performed using ICP multi-element standard solution VIII CertiPUR^®^ (Merck) and mono-elemental solutions for V and Ti (CertiPUR^®^, Merck). The concentrations of commercial standards concentration were 1 g L^−1^.

Standard Reference Material (SRM) samples were used. Particulate urban matter samples were provided by NIST (National Institute of Standards and Technology, Gaithersburg, MD, USA): The matrices of 1648 and 1648a NIST samples are similar to those of most of atmospheric airborne particles. Owing to the relative scarcity of atmospheric aerosol SRM samples, NIST SRM samples present a wide spectrum of certified metals, and are representative of the type of aerosol usually encountered in non-urban but non-remote coastal regions. Moreover, these SRM samples exhibit different solubilities [5]. The use of 100 mg of SRM is recommended for the purpose of method validation, but other studies have reported good and reproducible results with lower (5–10 mg) sample mass [1], [6]. Swami et al. [7] and Yang et al. [6] also reported that 0.5 mL of HF would be sufficient for complete recovery from silicate matrix samples (with using 5–10 mg of NIST 1648). We used in the present work 10 mg SRM. This amount can be likened to the average aerosol mass collected after a one-week sampling. We obtained very good precision with the small sample mass used throughout our recovery experiments (see *Validation method*). These SRM samples were used to define the mineralisation protocol offering the best compromise to digest a variety of trace elements (See comparative methods in Supplementary material).

SRM B-3 samples were also used. They were provided by the Department of Occupational Hygiene National Institute of Occupational Health, Oslo (Norway). Trace element concentrations on this filter correspond to the threshold limit value of contaminations in workroom atmospheres. Certified samples for aerosol are rare, and even more natural atmospheric matter SRM samples. SRM B-3 can therefore be used for chemical matrices found in unpolluted atmospheric aerosol samples.

The SRM sample TM-28.4 lot 0914 (Lake Ontario water) was provided by the National Research Council, Ottawa (Canada), and 1643e (artificial freshwater) was provided by the NIST. These two latter samples were used to design the proposed analytical method and control its accuracy.

Blank values were measured from cellulose acetate filters Sartorius SM 11106 (porosity 0.45 μ m, diameter 47 mm). Cellulose acetate filters were used as they completely digest in acidic mixtures, and because their metal content is compatible with trace metal analysis [4], [8].

To obtain the lowest blank values, filters were washed in a 5% hydrochloric acid bath for 2 h, rinsed with Milli-Q^®^ water and dried under a laminar flow. Because the filter may become brittle, it is recommended not to exceed a soaking time of more than 4 h.

All analyses were carried out under laminar airflow benches in a class-100 clean room.

The whole material was washed for 3 days in a 10% nitric acid bath, then rinsed with Milli-Q^®^ water and dried under a laminar flow hood.

### Hot block

A 54-Well HotBlock^®^ Environmental Express hot block was used. Its large capacity enables to digest up to 54 samples simultaneously, and it is made of corrosion-resistant graphite and Kydex^®^ to prevent metallic contamination. It is approved by the Environmental Protection Agency (EPA) and recommended to achieve EPA methods 3005 (water), 3010 (aqueous solution samples), 3050A and 3050B (sediment, sludge and soils), 200.2 and 200 (sample preparation). The heater mat provides uniform heat distribution to all samples throughout the digestion process, requiring less energy and radiating less heat than acid digestion with hot plates. The hot block maintains a setpoint of ±0.1 °C and permits sample consistency of ±1.5 °C. The stability of temperature allows all samples being digested to evaporate at a similar rate, providing better recoveries and preventing cross contaminations.

To better prevent contamination, the hot block was put inside the HEPA filtered AirLite hood.

All digestions were performed in 60 mL Teflon bottles (Nalgene^®^) overcome with a cap reflux condenser to promote a gentle refluxing.

### Inductively coupled plasma-optical emission spectrometer

Solutions of mineralised metals were analysed by a Perkin Elmer Optima 8000 DV inductively coupled plasma-optical emission spectrometry (ICP-OES). This instrument was equipped with a Meinhard^®^ concentric nebuliser type A3 and a cyclonic spray chamber that provide excellent sensibility and precision for aqueous solutions. A AS-90 series autosampler was used, which included a sample tray and a motorised sampling arm with attached Teflon^®^ probe. The entire system was controlled by winlab32 software.

The optimal operating conditions are listed in Table 1.

**Table 1:** ICP-OES PE optima 8000 DV operating conditions.

Sample introduction, plasma and data acquisition	
RF power	1450 W
Nebuliser gas flow	0.55 L min^−1^
Auxiliary gas flow	0.2 L min^−1^
Plasma gas flow	10 L min^−1^
Pump flow rate	1 mL min^−1^
Sample flow	1 mL min^−1^
Plasma view	Axial view
Peak processing	Peak area
Points per peak	5 pts
Plasma equilibration	10 s
Integration time	Min: 0.1 s Max: 5 s
Replicates	3

All calibrations and blanks were prepared in Milli-Q^®^ water acidified with nitric acid 65% to obtain a final solution of 2% nitric acid.

Calibration curves for Cd, Co, Ni, V, and As ranged from 5 to 100 μg/L, from 5 to 5000 μg/L for Ba, Cu, Mn, Ti, K, Mg, Na, Pb, and Zn, and from 5 to 10 000 μg/L for Al and Fe.

The calibration standards were stored in Teflon bottles. Linearity value for calibration curve of each metal was 1 > r^2^ > 0.99.

Low- and high- concentration standards (Reslope standards) were analysed after the calibration, and subsequently after every 10 samples to evaluate the drift of the instrument. If the drift was >5%, the instrument was calibrated again, and the last 10 samples were re-analysed. Also, a calibration check with SRM TM-28.4 lot 0914, and NIST 1643e was performed to evaluate the relative error.

## Procedure

All samples (natural aerosol samples + SRM samples) were dissolved by acid treatment by sets of 54 samples, including two blank filters, and two reagent blanks.

• 10 mg of NIST 1648 and 1648a were weighed on a filter with a Mettler Toledo XS205 precision balance.
• Each filter was folded into 4 and introduced into an acid-cleaned Teflon bottle (60 mL).
• The acid mixture was added: Aqua regia + HF (1.5 mL HCl 30% + 0.5 mL HNO_3_ 65% + 0.5 mL HF 40%).
• The set of bottles was put in ultrasonic bath during 10 min.
• Each Teflon bottle was placed in the block digester and overcome with a cap reflux condenser.
• Temperature programmes were set to 130 °C. To avoid the projection of sample particles, the temperature was progressively elevated: 2 °C min^−1^ to attain 130 °C and be maintained at this temperature during two hours.
• A residue was obtained and dissolved with 1.5 mL HNO_3_ (65%), ultrasonically agitated for 15 min, and made up to volume with 10 mL of Milli-Q^®^ water.
• The solution was finally stored in an acid-cleaned polypropylene tube (15 mL) and kept in the refrigerator at 4 °C until analysis.

Other tested protocols can be found in the *Supplementary material* section.

## Methods validation

### Validation of the mineralisation protocol

Digestion methods were validated by analysing SRM 1648 and 1648a samples.

Results from this mineralisation method agree well with the certified values (Table 2), except for the case of As in SRM 1648a (recovery: 84%). However, results are acceptable with SRM 1648 and the three SRM filters (recovery between 90 and 94%).

**Table 2:** Recovery of standard reference material SRM samples (n = 10). Numbers between parentheses are non-certified values (given for information by the NIST).

	SRM 1648			SRM 1648a				SRM B3-0561	SRM B3-0562	SRM B3-0563
Elements	Certified values	Units	Average of recovery (%)	Certified values	Units	Average of recovery (%)	Certified values (μg)	Average of recovery (%)	Average of recovery (%)	Average of recovery (%)
Al	*3.42* *±* *0.11*	%	97 ± 1	*3.43* *±* *0.13*	%	94 ± 1	*110* *±* *1*	97 ± 1	97 ± 1	96 ± 2
As	*115* *±* *10*	mg kg^−1^	94 ± 7	*115.5* *±* *3.9*	mg kg^−1^	84 ± 7	*3.76* *±* *0.04*	90 ± 4	92 ± 2	91 ± 1
Ba	*(737)*	mg kg^−1^	92 ± 3	–	–	–	*18.4* *±* *0.2*	96 ± 1	95 ± 1	97 ± 1
Cd	*75* *±* *7*	mg kg^−1^	102 ± 1	*73.7* *±* *2.3*	mg kg^−1^	95 ± 1	*7.35* *±* *0.07*	98 ± 2	95 ± 2	96 ± 1
Co	*(18)*	mg kg^−1^	107 ± 5	*17.93* *±* *0.68*	mg kg^−1^	100 ± 5	*18.3* *±* *0.2*	100 ± 1	104 ± 2	100 ± 1
Cu	*609* *±* *27*	mg kg^−1^	103 ± 1	*610* *±* *70*	mg kg^−1^	98 ± 1	*36.9* *±* *0.4*	97 ± 1	101 ± 1	97 ± 2
Fe	*3.91* *±* *0.1*	%	97 ± 1	*3.92* *±* *0.21*	%	93 ± 1	*256* *±* *3*	95 ± 1	95 ± 1	96 ± 2
K	*1.05* *±* *0.01*	%	97 ± 1	*1.056* *±* *0.049*	%	97 ± 1	*–*	–	–	–
Mg	*(0.8)*	%	98 ± 5	*0.813* *±* *0.012*	%	96 ± 5	*36.6* *±* *0.4*	96 ± 1	96 ± 1	95 ± 4
Mn	*786* *±* *17*	mg kg^−1^	100 ± 1	*790* *±* *44*	mg kg^−1^	94 ± 1	*73.6* *±* *0.8*	98 ± 2	97 ± 4	99 ± 3
Na	*0.425* *±* *0.002*	%	94 ± 3	*4240* *±* *60*	mg kg^−1^	95 ± 3	*–*	–	–	–
Ni	*82* *±* *3*	mg kg^−1^	101 ± 1	*81.1* *±* *6.8*	mg kg^−1^	97 ± 1	*29.7* *±* *0.3*	99 ± 1	100 ± 2	102 ± 1
Pb	*0.655* *±* *0.008*	%	101 ± 2	*0.655* *±* *0.033*	%	95 ± 1	*18.2* *±* *0.2*	94 ± 2	93 ± 2	92 ± 1
Ti	*(0.4)*	%	91 ± 2	*4021* *±* *86*	mg kg^−1^	92 ± 2	*17.8* *±* *0.4*	99 ± 1	98 ± 1	99 ± 1
V	*127* *±* *7*	mg kg^−1^	103 ± 2	*127* *±* *11*	mg kg^−1^	90 ± 2	*7.61* *±* *0.08*	98 ± 1	96 ± 2	97 ± 2
Zn	*0.479* *±* *0.014*	%	103 ± 1	*4800* *±* *270*	mg kg^−1^	98 ± 1	*111* *±* *1*	98 ± 3	96 ± 1	97 ± 1

### Validation of the analytical method

Theoretical detection limits (defined as three times the standard deviation of the blank) and quantitation limits (defined as ten times the standard deviation of the blank) were calculated by analysing the blank samples and were expressed in μg L^−1^ for each element (Table 3).

**Table 3:** Wavelengths, blanks, detection and quantification limits.

Elements	λ (nm)	Average filter blank (μg L^−1^) n = 10	Average reagent blank (μg L^−1^) n = 10	Detection limit (μg L^−1^)	Quantification limit (μg L^−1^)
Al	396.153	9.60	3.30	1.01	3.35
As	197.197	4.98	2.98	5.32	17.72
Ba	455.403	11.41	5.66	0.03	0.09
Cd	228.802	2.80	1.87	0.41	1.36
Co	228.616	2.79	1.66	0.58	1.93
Cu	327.393	7.05	0.28	0.79	2.65
Fe	238.204	4.23	1.38	0.39	1.29
K	766.490	35.62	34.84	1.20	4.00
Mg	285.213	14.84	3.68	0.20	0.65
Mn	257.610	3.40	2.28	0.03	0.12
Na	589.592	41.33	30.66	0.69	2.29
Ni	231.604	5.08	2.18	0.75	2.51
Pb	220.353	5.80	4.80	1.69	5.62
Ti	334.940	3.91	3.60	0.09	0.30
V	290.880	10.44	8.11	0.60	2.01
Zn	206.200	7.73	3.72	0.30	0.99

The detection limits were <1 μg L^−1^ for most metals except Al, K and Pb (<2 μg L^−1^), and As (5.32 μg L^−1^).

The precision of the method was evaluated by measuring NIST 1643e (n = 10) and TM-28.4 for Ti. The%RSD were 1.5 (Al), 1.4 (As), 0.6 (Ba), 5.8 (Cd), 2.35 (Co), 3.43 (Cu), 1.3 (Fe), 1.9 (K), 0.1 (Mg), 0.7 (Mn), 1.5 (Na), 0.9 (Ni), 1.41 (Pb), 5.4 (Ti), 1.8 (V), and 1 (Zn).

The analytical procedure was checked by analysing two SRM samples: TM-28.4 lot 0914 and NIST 1643e. Table 4 shows the average recovery observed between certified and measured values.

**Table 4:** Validation of the analytical method, using standard reference material (SRM) samples. Average measured values were calculated from 10 replicates.

		NIST 1643e		TM-28.4	
Elements	λ (nm)	Certified (μg L^−1^)	Measured (average) (μg L^−1^)	Certified (μg L^−1^)	Measured (average) (μg L^−1^)
Al	396.153	141.8 ± 8.6	141.8 ± 1.5	54.3 ± 6.07	48 ± 4.3
As	197.197	60.45 ± 0.72	59.6 ± 0.86	6.27 ± 0.558	6.4 ± 1.3
Ba	455.403	544.2 ± 5.8	546.5 ± 3.7	16 ± 1.05	16.3 ± 0.4
Cd	228.802	6.568 ± 0.073	6.8 ± 0.4	1.91 ± 0.146	2.1 ± 0.1
Co	228.616	27.06 ± 0.32	26.3 ± 0.62	3.54 ± 0.70	4.8 ± 0.5
Cu	327.393	20.40 ± 0.24	20.4 ± 0.7	4.90 ± 0.381	5 ± 0.4
Fe	238.204	22.76 ± 0.31	22.1 ± 0.3	6.52 ± 0.730	5.9 ± 0.5
K	766.490	98.1 ± 1.4	100.7 ± 2.0	17.8 ± 3.11	18.2 ± 0.6
Mg	285.213	2034 ± 29	2040.9 ± 2.4	–	–
Mn	257.610	8037 ± 98	7925.6 ± 62.1	–	–
Na	589.592	38.97 ± 0.45	37 ± 0.56	6.96 ± 0.450	6.6 ± 2.6
Ni	231.604	20740 ± 260	21022.9 ± 200.0	–	–
Pb	220.353	62.41 ± 0.69	63.6 ± 0.9	9.87 ± 0.944	10.7 ± 2.4
Ti	334.940	–	–	8.13 ± 0.694	8.6 ± 1.35
V	290.880	37.86 ± 0.59	37.5 ± 0.7	3.18 ± 0.324	3.2 ± 0.2
Zn	206.200	78.5 ± 2.2	78.6 ± 0.8	29.5 ± 3.71	30.7 ± 0.7

## Conclusion

The aim of the present work was to propose an alternative mineralisation protocol, adapted to the analysis of major and trace metals in atmospheric aerosol samples, permitting the complete digestion of refractory and labile metals at the same time and without loss, and using an ICP-OES spectrometer. Advantages of the proposed protocol are mainly easiness, reliability, possibility to digest an important number of samples at the same time (with using a hot block), reduction of costs (notably because of the use of hot block, and the use of ICP-OES), and better accordance with Occupational Health and Safety requirements, owing to the reduced use of acids, in particular HF. This protocol is now used routinely in the monitoring of atmospheric deposition of trace metals at North-western Mediterranean time-series stations in the framework of the Mediterranean Ocean Observing System for Environment (MOOSE), DOI: 10.6096/MOOSE.762 [9].
