# Supplementary material for: Mineralisation of atmospheric aerosol particles and further analysis of trace elements by inductively coupled plasma-optical emission spectrometry
Source: MethodsX. 2017 Jun 13;4:191–8. doi: 10.1016/j.mex.2017.05.002 (PMC5480268; doi:10.1016/j.mex.2017.05.002)
Supplement: Supplementary file 1 [file mmc1.docx]

***Supplementary material and additional information***

To develop this protocol, four methodologies were evaluated on SRM 1648 and SRM 1648a:

Method 1: Aqua regia (2 mL HCl + 1 mL HNO_3_);

Method 2: Aqua regia + HF (1.5 mL HCl + 0.5 mL HNO_3_ + 0.5 mL HF);

Method 3: 3 mL HNO_3_;

Method 4: 2.5 mL HNO_3_ + 0.5 mL HF.

For each methodology, three temperature programmes were evaluated: 90, 130, and 160°C (See Tables 5a and b).

| Elements | SRM 1648  Certified values | Method 1  HNO_3_/HCl | | |  | Method 2  HNO_3_/HCl/HF | | |  | Method 3  HNO_3_ | | |  | Method 4  HNO_3_/HF | | | |
| --- | --- | --- | --- | --- | --- | --- | --- | --- | --- | --- | --- | --- | --- | --- | --- | --- | --- |
|  |  | **90°C** | **130°C** | **160°C** |  | **90°C** | **130°C** | **160°C** |  | **90°C** | **130°C** | **160°C** |  | **90°C** | **130°C** | **160°C** |  |
| Al | *3.42 ± 0.11* | 30 ± 1 | 31 ± 4 | 28 ± 2 |  | 94 ± 2 | 97 ± 1 | 89 ± 3 |  | 25 ± 1 | 29 ± 2 | 21 ± 3 |  | 90 ± 6 | 95 ± 1 | 94 ± 1 |  |
| As | *115 ± 10* | 96 ± 8 | 94 ± 9 | 74 ± 12 |  | 93 ± 6 | 94 ± 7 | 73 ± 6 |  | 97 ± 6 | 96 ± 7 | 79 ± 3 |  | 92 ± 8 | 90 ± 3 | 64 ± 5 |  |
| Ba | *(737)* | 51 ± 7 | 67 ± 7 | 72 ± 5 |  | 53 ± 9 | 92 ± 3 | 99 ± 4 |  | 41 ± 6 | 42 ± 9 | 66 ± 2 |  | 70 ± 7 | 82 ± 8 | 97 ± 3 |  |
| Cd | *75 ± 7* | 101 ± 3 | 108 ± 7 | 110 ± 4 |  | 99 ± 3 | 102 ± 1 | 103 ± 3 |  | 106 ± 5 | 107 ± 5 | 109 ± 1 |  | 105 ± 11 | 105 ± 2 | 102 ± 1 |  |
| Co | *(18)* | 86 ± 4 | 93 ± 10 | 109 ± 5 |  | 101 ± 2 | 107 ± 5 | 100 ± 13 |  | 91 ± 3 | 93 ± 8 | 104 ± 3 |  | 107 ± 9 | 109 ± 5 | 97 ± 5 |  |
| Cu | *609 ± 27* | 104 ± 4 | 101 ± 6 | 102 ± 6 |  | 101 ± 4 | 103 ± 1 | 102 ± 2 |  | 102 ± 3 | 103 ± 2 | 100 ± 1 |  | 105 ± 8 | 101 ± 1 | 99 ± 3 |  |
| Fe | *3.91 ± 0.1* | 74 ± 4 | 74 ± 5 | 67 ± 4 |  | 94 ± 3 | 97 ± 1 | 88 ± 3 |  | 59 ± 3 | 64 ± 3 | 56 ± 1 |  | 93 ± 8 | 95 ± 2 | 89 ± 0.4 |  |
| K | *1.05 ± 0.01* | 22 ± 1 | 23 ± 2 | 23 ± 2 |  | 95 ± 1 | 97 ± 1 | 92 ± 1 |  | 21 ± 1 | 22 ± 1 | 21 ± 1 |  | 65 ± 5 | 63 ± 1 | 60 ± 1 |  |
| Mg | *(0.8)* | 78 ± 2 | 81 ± 5 | 70 ± 6 |  | 89 ± 5 | 98 ± 5 | 95 ± 1 |  | 76 ± 1 | 80 ± 2 | 67 ± 2 |  | 92 ± 8 | 93 ± 3 | 92 ± 2 |  |
| Mn | *786 ± 17* | 95 ± 6 | 94 ± 5 | 89 ± 5 |  | 99 ± 3 | 100 ± 1 | 100 ± 1 |  | 89 ± 5 | 91 ± 3 | 85 ± 3 |  | 106 ± 4 | 104 ± 1 | 130 ± 4 |  |
| Na | *0.425 ± 0.002* | 40 ± 2 | 39 ± 3 | 41 ± 2 |  | 95 ± 2 | 94 ± 3 | 86 ± 2 |  | 38 ± 1 | 38 ± 1 | 37 ± 1 |  | 96 ± 10 | 92 ± 3 | 86 ± 2 |  |
| Ni | *82 ± 3* | 90 ± 4 | 96 ± 9 | 135 ± 5 |  | 102 ± 2 | 101 ± 1 | 144 ± 4 |  | 88 ± 3 | 96 ± 4 | 114 ± 5 |  | 115 ± 8 | 114 ± 1 | 125 ± 3 |  |
| Pb | *0.655 ± 0.008* | 100 ± 4 | 103 ± 6 | 90 ± 6 |  | 93 ± 3 | 101 ± 2 | 93 ± 3 |  | 99 ± 4 | 95 ± 5 | 90 ± 2 |  | 91 ± 7 | 101 ± 2 | 100 ± 1 |  |
| Ti | *(0.4)* | 11 ± 1 | 12 ± 2 | 12 ± 1 |  | 77 ± 2 | 91 ± 2 | 96 ± 1 |  | 7 ± 1 | 8 ± 1 | 8 ± 1 |  | 80 ± 5 | 83 ± 1 | 91 ± 3 |  |
| V | *127 ± 7* | 107 ± 3 | 102 ± 13 | 105 ± 7 |  | 102 ± 3 | 103 ± 2 | 109 ± 6 |  | 106 ± 3 | 104 ± 6 | 100 ± 5 |  | 105 ± 10 | 105 ± 2 | 125 ± 5 |  |
| Zn | *0.479 ± 0.014* | 98 ± 3 | 101 ± 6 | 92 ± 7 |  | 96 ± 3 | 103 ± 1 | 96 ± 4 |  | 97 ± 4 | 100 ± 1 | 92 ± 2 |  | 94 ± 11 | 93 ± 2 | 94 ± 1 |  |

Table 5a: Recovery of standard reference material (SRM) samples (NIST 1648). Concentrations are expressed in μg g^-1^ or in % w/w (Al, Fe, K, Mg, Na, Pb, Ti, Zn). The average recovery observed between certified and measured values is expressed in %. Numbers between parentheses are non-certified values (given for information by the NIST).

| Elements | SRM 1648a  Certified values | | Method 1  HNO_3_/HCl | | | | |  | Method 2  HNO_3_/HCl/HF | | |  | | | Method 3  HNO_3_ | | | Method 4  HNO_3_/HF | | |
| --- | --- | --- | --- | --- | --- | --- | --- | --- | --- | --- | --- | --- | --- | --- | --- | --- | --- | --- | --- | --- |
|  | |  | | **90°C** | **130°C** | **160°C** |  | **90°C** | | **130°C** | **160°C** | |  | **90°C** | | **130°C** | **160°C** | **90°C** | **130°C** | **160°C** |
| Al | | *3.43 ± 0.13* | | 42 ± 1 | 45 ± 4 | 41 ± 2 |  | 89 ± 2 | | 94 ± 1 | 95 ± 3 | |  | 18 ± 1 | | 48 ± 2 | 63 ± 3 | 86 ± 6 | 91 ± 1 | 93 ± 1 |
| As | | *115.5 ± 3.9* | | 92 ± 8 | 97 ± 9 | 72 ± 12 |  | 93 ± 6 | | 84 ± 7 | 70 ± 6 | |  | 91 ± 6 | | 107 ± 7 | 71 ± 3 | 93 ± 8 | 85 ± 3 | 73 ± 5 |
| Cd | | *73.7 ± 2.3* | | 93 ± 3 | 96 ± 7 | 92 ± 4 |  | 91 ± 3 | | 95 ± 1 | 85 ± 3 | |  | 93 ± 5 | | 108 ± 5 | 91 ± 1 | 88 ± 11 | 102 ± 2 | 85 ± 1 |
| Co | | *17.93 ± 0.68* | | 91 ± 4 | 93 ± 10 | 93 ± 5 |  | 89 ± 2 | | 100 ± 5 | 91 ± 13 | |  | 92 ± 3 | | 79 ± 8 | 86 ± 3 | 91 ± 9 | 89 ± 5 | 92 ± 5 |
| Cu | | *610 ± 70* | | 90 ± 4 | 91 ± 6 | 92 ± 6 |  | 96 ± 4 | | 98 ± 1 | 95 ± 2 | |  | 93 ± 3 | | 96 ± 2 | 95 ± 1 | 97 ± 8 | 100 ± 1 | 98 ± 3 |
| Fe | | *3.92 ± 0.21* | | 61 ± 4 | 74 ± 5 | 75 ± 4 |  | 87 ± 3 | | 93 ± 1 | 95 ± 3 | |  | 58 ± 3 | | 91 ± 3 | 79 ± 1 | 86 ± 8 | 91 ± 2 | 95 ± 1 |
| K | | *1.056 ± 0.049* | | 30 ± 1 | 38 ± 2 | 22 ± 2 |  | 86 ± 1 | | 97 ± 1 | 91 ± 1 | |  | 30 ± 1 | | 78 ± 1 | 62 ± 1 | 60 ± 5 | 61 ± 1 | 68 ± 1 |
| Mg | | *0.813 ± 0.012* | | 85 ± 2 | 90 ± 5 | 92 ± 6 |  | 82 ± 5 | | 96 ± 5 | 89 ± 1 | |  | 79 ± 3 | | 91 ± 2 | 81 ± 2 | 84 ± 8 | 90 ± 3 | 89 ± 2 |
| Mn | | *790 ± 44* | | 81 ± 6 | 81 ± 5 | 82 ± 5 |  | 78 ± 3 | | 94 ± 1 | 92 ± 1 | |  | 81 ± 5 | | 96 ± 3 | 96 ± 3 | 89 ± 4 | 87 ± 1 | 99 ± 4 |
| Na | | *4240 ± 60* | | 53 ± 2 | 59 ± 3 | 51 ± 2 |  | 90 ± 2 | | 95 ± 3 | 87 ± 2 | |  | 39 ± 1 | | 66 ± 3 | 69 ± 1 | 80 ± 10 | 86 ± 3 | 93 ± 2 |
| Ni | | *81.1 ± 6.8* | | 80 ± 4 | 92 ± 9 | 92 ± 5 |  | 94 ± 2 | | 97 ± 1 | 96 ± 4 | |  | 68 ± 3 | | 95 ± 4 | 112 ± 5 | 91 ± 8 | 96 ± 1 | 114 ± 3 |
| Pb | | *0.655 ± 0.033* | | 89 ± 4 | 96 ± 6 | 83 ± 6 |  | 93 ± 3 | | 95 ± 1 | 85 ± 3 | |  | 94 ± 4 | | 99 ± 5 | 89 ± 2 | 93 ± 7 | 93 ± 2 | 81 ± 1 |
| Ti | | *4021 ± 86* | | 11 ± 1 | 17 ± 2 | 15 ± 1 |  | 82 ± 2 | | 92 ± 2 | 96 ± 1 | |  | 7 ± 1 | | 56 ± 1 | 18 ± 1 | 87 ± 5 | 89 ± 1 | 97 ± 3 |
| V | | *127 ± 11* | | 89 ± 3 | 85 ± 13 | 89 ± 7 |  | 87 ± 3 | | 90 ± 2 | 88 ± 6 | |  | 84 ± 3 | | 74 ± 6 | 90 ± 5 | 90 ± 10 | 83 ± 2 | 91 ± 5 |
| Zn | | *4800 ± 270* | | 95 ± 3 | 96 ± 6 | 92 ± 7 |  | 91 ± 3 | | 98 ± 1 | 97 ± 4 | |  | 83 ± 4 | | 97 ± 1 | 96 ± 2 | 91 ± 11 | 93 ± 2 | 90 ± 1 |

Table 5b: Recovery of standard reference material (SRM) samples (NIST 1648a). Concentrations are expressed in μg g^-1^ or in % w/w (Al, Fe, K, Mg, Pb). The average recovery observed between certified and measured values is expressed in %.

*SRM sample analysis with and without HF*

The use of HF is generally necessary to dissolve alumino-silicates [10]. The use of aqua regia is permitted with ICP-OES spectrophotometers, but it is more problematic with inductively coupled plasma-mass spectrometers (ICP-MS), due to the presence of chloride ions, which may cause polyatomic isobaric interferences [6]. Aqua regia is considered more efficient than HNO_3_ alone for very reducing metals that require a strong oxidising agent, to which an acidic agent (HCl) is added. The efficiency of four mineralisation procedures was tested using the SRM samples NIST 1648 and NIST 1648a. Each batch of SRM samples included two reagent blank samples and two filter blank samples. All results are presented in Table 5a and 5b.

Method 1 (Aqua regia only) is satisfactory even at 90°C for the two SRM samples, for metals such as As, Cd, Co, Cu, Mn Ni, V, and Zn, i.e. soluble to moderately soluble metals. The increase of temperature may be a way to improve digestion (e.g. Co), but it may also lead to some loss of metal (e.g. As and Pb at 160°C. This was observed for the case of As everytime at this temperature, whatever the programme). Digestion is moderately satisfactory for Mn in NIST 1648a. For insoluble metals such as Al, Ba, Fe, and Ti, however, digestion is clearly insufficient, and the increase of temperature does not change significantly the efficiency of digestion. Among the lowest recoveries are K and Na, and, to a much lesser extent (and only for NIST 1648a), Mg when only aqua regia is used, despite the high solubility of these metals. The addition of HF significantly increases the efficiency of digestion. This peculiarity has been already observed by Ventura et al.[11]. This suggests that these metals either occur as part of insoluble minerals such as phyllosilicates, or a fraction of them is embedded by other insoluble material, and, therefore, protected from acids other than HF. Peng et al. [12] have shown that dust produced in sintering production and iron and steel metallurgy may comprise such insoluble forms of K and Na in residual dust particles, which partly protects soluble K and Na chlorides.

Method 2 (aqua regia + HF) appears more efficient, at least if the lowest temperature is discarded (Ba and Ti). As mentioned above, the addition of HF permits good recoveries for K, Na and Mg. Method 3 (HNO_3_ only) should be discarded for insoluble metals, and also for K, Na, and Mg, whatever the temperature. The fourth method (HNO_3_ + HF) seems fairly good for most of insoluble metals at 130°C, except Ba and Ti, for both SRM samples. The digestion of Mn and V is not satisfactory for NIST 1648a, again. In addition, a refractory part of K remains undigested whatever the temperature, while Na and Mg can be totally digested. It seems that a slight part of Na is lost at 160°C, however.

These comparisons suggest that the protocol 2 (mixture aqua regia/HF) is the most all-purpose option. Partial losses of metals may occur at 160°C (e.g. As, and possibly Pb, too). On the contrary, a temperature of 90°C does not appear to be high enough to totally digest metals such as Ba and Ti (SRM 1648 and 1648a), and Ba, Ti, Mn and Mg (SRM 1648a). The best compromise for this protocol is 130°C, although a slight loss of As (9%) was still observed in SRM 1648a, even at 130°C. No loss was noticed for SRM 1648.

The efficiency of digestions carried out here with the mixture aqua regia/HF is compared with that of other methods for the same metals, using the same SRM samples ([3], [8], [11], [13]–[16]Tables 6a and b).

| Elements | SRM 1648 Certified values | This study | Celo et al.  [8] | | Kulkarni et al  [13] | Toscano et al  [14] |
| --- | --- | --- | --- | --- | --- | --- |
|  |  | HNO_3_/HCl/  HF | 40% (v/v) HNO_3_ | HF/  HNO_3_/  H_3_BO_3_ | HNO_3_/  HF/  H_3_BO_3_ | HNO_3_/  HF |
| Al | *3.42 ± 0.11* | 97 ± 1 | 50 ± 1 | 86 ± 10 | 104 ± 8 | 91 ± 2 |
| As | *115 ± 10* | 94 ± 7 | 104 ± 8 | 102 ± 4 | 109 ± 7 | 91 ± 3 |
| Ba | *(737)* | 92 ± 3 | 82 ± 8 | 102 ± 10 | 101 ± 3 | - |
| Cd | *75 ± 7* | 102 ± | 107 ± 7 | 99 ± 5 | 112 ± 4 | 89 ± 3 |
| Co | *(18)* | 107 ± 5 | 81 ± 5 | 90 ± 6 | 98 ± 8 | - |
| Cu | *609 ± 27* | 103 ± 1 | 97 ± 6 | 91 ± 5 | 91 ± 7 | 97 ± 5 |
| Fe | *3.91 ± 0.1* | 97 ± 1 | 82 ± 11 | 92 ± 10 | 98 ± 4 | 102 ± 10 |
| K | *1.05 ± 0.01* | 97 ± 1 | - | - | - | - |
| Mg | *(0.8)* | 98 ± 5 | - | - | - | - |
| Mn | *786 ± 17* | 100 ± 1 | 94 ± 8 | 99 ± 7 | 96 ± 5 | 97 ± 5 |
| Na | *0.425 ± 0.002* | 94 ± 3 | - | - | - | - |
| Ni | *82 ± 3* | 101 ± 1 | 89 ± 7 | 93 ± 10 | 99 ± 4 | 83 ± 4 |
| Pb | *0.655 ± 0.008* | 101 ± 2 | 101 ± 9 | 98 ± 4 | 108 ± 5 | 96 ± 2 |
| Ti | *(0.4)* | 91 ± 2 | 28 ± 2 | 73 ± 10 | 107 ± 4 | - |
| V | *127 ± 7* | 103 ± 2 | 83 ± 7 | 96 ± 6 | 101 ± 6 | 87 ± 3 |
| Zn | *0.479 ± 0.014* | 103 ± 1 | 116 ± 9 | 94 ± 4 | 101 ± 9 | 120 ± 4 |

Table 6a: Recoveries (%) of present metal analyses using protocol 2 (aqua regia + HF) at 130°C compared with other published results. All studies used the same SRM sample (NIST 1648). Concentrations of SRM 1648 are expressed in μg g^-1^ or in % w/w (Al, Fe, K, Mg, Na, Pb, Ti, Zn).

| Elements | SRM 1648a Certified values | This study | Salcedo et al.  [14] | Ventura et al.  [11] | De Paula et al  [16] | Da Silva et al.  [3] |
| --- | --- | --- | --- | --- | --- | --- |
|  |  | HNO_3_/HCl/  HF | HNO_3_ | HNO_3_/  HF | HNO_3_ | HNO_3_/  HCl |
| Al | *3.43 ± 0.13* | 94 ± 1 | 17 | 77 | - | - |
| As | *115.5 ± 3.9* | 84 ± 7 | - | 88 | - | - |
| Cd | *73.7 ± 2.3* | 95 ± 1 | 83 | 88 | 92 | 81 |
| Co | *17.93 ± 0.68* | 100 ± 5 | - | 79 | - | 83 |
| Cu | *610 ± 70* | 98 ± 1 | 93 | 78 | 81 | 103 |
| Fe | *3.92 ± 0.21* | 93 ± 1 | 58 | 82 | 63 | 100 |
| K | *1.056 ± 0.049* | 97 ± 1 | 30 | - | 49 | - |
| Mg | *0.813 ± 0.012* | 96 ± 5 | 69 | 69 | 74 | - |
| Mn | *790 ± 44* | 94 ± 1 | 81 | 87 | 61 | 100 |
| Na | *4240 ± 60* | 95 ± 3 | 39 | - | - | - |
| Ni | *81.1 ± 6.8* | 97 ± 1 | 68 | 102 | - | 93 |
| Pb | *0.655 ± 0.033* | 95 ± 2 | 94 | 87 | 98 | 86 |
| Ti | *4021 ± 86* | 92 ± 2 | 7 | 84 | 7 | - |
| V | *127 ± 11* | 90 ± 2 | 64 | 80 | - | 92 |
| Zn | *4800 ± 270* | 98 ± 1 | 83 | 87 | 81 | 108 |

Table 6b: Recoveries (%) of present metal analyses using protocol 2 (aqua regia + HF) at 130°C compared with other published results. All studies used the same SRM sample (NIST 1648a). Concentrations of SRM 1648a are expressed in μg g^-1^ or in % w/w (Al, Fe, K, Mg, Pb).

This comparison suggests that the current method results in satisfactory digestions (except for As), i.e. its efficiency is equivalent to that of other studies cited in Table 6a and 6b, and, in a number of cases, significantly better (e.g. insoluble metals such as Ti or Al, but also soluble metals such as K or Na, as mentioned above). This applies to both NIST 1648 and NIST 1648a samples. In all cases, the use of HF is necessary, at minimum for insoluble metals usually found in mineral matrices, e.g. Saharan dust material (Al, Fe, Mn, Ti…). It is noteworthy that most of the studies mentioned in Table 6 ([3], [8], [11], [13]–[16]) propose microwave-assisted mineralisation protocols. The use of hot block does not lower the efficiency of digestion, as shown by this comparison. It should be also reminded that only small amounts of sample (10 mg, typically) are needed here.

All the mineralisation protocols tested here permit further ICP-OES analyses, while the use of aqua regia may be problematic for, e.g., ICP-MS measurements, as already mentioned. Apart from the case of ultra-trace measurements [17]. (see quantification limits in Table 3), which rarely applies to standard atmospheric aerosol samples, and apart from some specific metals (e.g. platinum group elements), ICP-OES detection limits, standard deviations and reproducibility are very acceptable, as shown by Tables 6a and b. Studies [3], [8], [13]–[15] cited in Tables 5a and b used an ICP-MS spectrometer. De Paula et al. [16] have used it only for trace metals, and used an ICP-OES spectrometer for major metals.

Atmospheric aerosol samples can thus be completely digested with using hot blocks, and further analyses can be carried out with an ICP-OES spectrometer. This should be taken into consideration, e.g. for cost reasons.
